# Metabolic Rate and Oxidative Stress as a Risk Factors in the Development of Colorectal Cancer

**DOI:** 10.3390/ijms251910713

**Published:** 2024-10-05

**Authors:** Diana Sawicka, Sebastian Maciak, Anna Sadowska, Emilia Sokołowska, Sylwia Gohal, Katarzyna Guzińska-Ustymowicz, Katarzyna Niemirowicz-Laskowska, Halina Car

**Affiliations:** 1 Department of Experimental Pharmacology, Medical University of Bialystok, Szpitalna Street 37, 15-295 Bialystok, Poland; anna.sadowska@umb.edu.pl (A.S.); zfarmdosw@umb.edu.pl (S.G.); katarzyna.niemirowicz-laskowska@umb.edu.pl (K.N.-L.); halina.car@umb.edu.pl (H.C.); 2 Department of Evolutionary and Physiological Ecology, Faculty of Biology, University of Bialystok, Ciolkowskiego Street 1J, 15-245 Bialystok, Poland; maciaks@uwb.edu.pl; 3 Department of Clinical Pharmacology, Medical University of Bialystok, Waszyngtona Street 15A, 15-274 Bialystok, Poland; emiliasokolowska.umwb@gmail.com; 4 Department of General Pathomorphology, Medical University of Bialystok, Waszyngtona Street 13, 15-269 Bialystok, Poland; katarzyna.guzinska-ustymowicz@umb.edu.pl

**Keywords:** metabolic rate, colorectal cancer, oxidative stress, keap1

## Abstract

There is growing evidence that the body’s energy expenditures constitute a significant risk factor for the development of most deadly diseases, including cancer. Our aim was to investigate the impact of basal metabolic rate (BMR) on the growth and progression of colorectal cancer (CRC). To do so, we used a unique model consisting of three lines of laboratory mice (*Mus musculus*) artificially selected for high (HBMR) and low (LBMR) basal metabolic rate and randomly bred individuals (non-selected, NSBMR). The experimental individuals were implanted with human colorectal cancer cells DLD-1. The variation in BMR between the lines allowed for testing the impact of whole-body metabolism on oxidative and antioxidant parameters in the liver throughout the cancerogenesis process. We investigated the dependence between metabolic values, reactive oxygen species (ROS) levels, and Kelch-like ECH-associated protein 1-based E3 ligase complexes (*Keap1*) gene activity in these animals. We found that the HBMR strain had a higher concentration of oxidative enzymes compared to the LBMR and NSBMR. Furthermore, the growth rate of CRC tumors was associated with alterations in the levels of oxidative stress enzymes and *Keap1* expression in animals with a high metabolic rate. Our results indicate that a faster growth and development of CRC line DLD-1 is associated with enzymatic redox imbalance in animals with a high BMR.

## 1. Introduction

Colorectal cancer is one of the most common malignant tumors in the world. About one million people are diagnosed with CRC each year, resulting in 8% of cancer deaths [1]. However, the exact molecular mechanisms responsible for tumor development remain unclear. Although studies concerning CRC continue to search for a universal mechanism underlying overall cancer risk, suggesting the recruitment of additional tumor suppressor genes or the enhanced activation of the immune system, still, they have brought minor advancement in clinical trials [2,3]. Therefore, understanding the genetic and physiological mechanisms of cancer initiation and its progression could be essential for future preventive and therapeutic approaches. 

Organismal energy expenditures constitute a significant factor contributing to the risk of developing the currently most deadly diseases, including cancer [4,5]. The high energy turnover is associated with lipid membrane peroxidation, negatively affecting multiple cellular functions, like DNA modification, single-strand breaks, and protein carbonylation that cause enzymatic inactivation and increase the likelihood of proteolysis [6]. Cancer cells are characterized by their limitless replication potential, which is associated with a high energy requirement [7]. It has been demonstrated that energy metabolic dysfunctions have a significant impact on the activity of oncogenes, tumor suppressors, and mitochondrial DNA mutations via specific signaling pathways. Thus, a strong relationship between cancers and energy metabolism most probably exists. The commonly used measurement of metabolic rate (MR) is a basal metabolic rate, which quantifies the minimum rate of energy expenditure necessary to maintain the efficiency balance of a resting, post-absorptive state at thermoneutral conditions [8]. The increase in MR is expected to alter DNA replication, as well as the methylation of histone proteins, that are involved in angiogenesis [9,10]. To date, it is broadly accepted that the level of ROS production in an organism is a function of basal metabolic rate [4,5]. Thus, any disturbance in MR should be associated with oxidative stress, which may be caused by an increase in free radical generation and/or a decrease in antioxidant levels in the target cells and tissues. Presently, many epidemiologic studies support the role of reactive oxygen radicals in the development of cancer and the protective role of antioxidant enzymes against tumor formation. Oxidative stress is also involved in genetic and epigenetic changes in CRC development [11,12]. The relationship between metabolic overload, free radical generation, and the probability of cancer development seems unquestionable, but there is still a lack of empirical data to determine the exact mechanisms responsible for tumor initiation. 

In order to understand the processes of carcinogenesis, it is crucial to conduct studies at the organismal level that enable a comprehensive analysis of cancer-related traits within a species. Experiments based on artificial models that emulate the action of natural selection provide a robust test of the associations between metabolic rates and the likelihood of cancer development [13]. Here, we used a specific model consisting of three lines of laboratory mice (*Mus musculus*): artificially selected for high and low basal metabolic rate and randomly bred individuals, non-selected, line), which were implanted with the human colorectal cancer cells DLD-1 applied to in vivo models of CRC development [14,15,16,17,18]. HBMR individuals are characterized by considerably higher energy expenditures, which are tightly linked to greater oxygen consumption, an increase in food intake, and changes in body composition, exemplifying over 50% differentiation in BMR between selected lines [19]. Moreover, suggested representatives differ significantly in the key traits directly related to carcinogenesis, including immune response, intoxication rate, or oxidative damage [13,20]. Thus, between-line variation in BMR allowed us to test for the impact of whole-body metabolism on the probability and the development rate of cancerogenic changes. We examined the total oxidative and antioxidant status in the livers of those animals as the markers of metabolic load. Hepatocytes constitute a key metabolic tissue that regulates energy turnover and serves as the primary detoxifying organ, maintaining metabolic homeostasis and metabolizing various compounds that produce ROS [21]. The occurrence of oxidative stress is often a critical factor for intracellular signaling molecules that control cellular functions, such as proliferation, differentiation, growth, and apoptosis [22]. Potential defense mechanisms defined to counteract ROS damage include specific genes encoding detoxification enzymes and proteins. Nuclear factor erythroid-related factor 2 (Nrf2) is responsible for regulating the key signaling pathways that maintain cellular redox homeostasis in the liver. Under physiological conditions, *Nrf2* is ubiquitinated by *Keap1* in the cytosol and is degraded through the proteasome [23]. 

The aim of this study was to verify whether the level of cell proliferation in induced CRC tumors of the DLD-1 line is dependent on BMR, as well as to check the impact of genetically determined metabolism and oxidation/antioxidation status on CRC growth rate. In addition, we also tested the activity of the *Keap1* gene under conditions of oxidative stress and its role in the regulation of the antioxidant response in animals with different metabolic rates.

## 2. Results and Discussion

### 2.1. BMR Values and Body Mass

There is a growing consensus that metabolic overload can significantly contribute to the risk of colorectal cancer [24]. Accordingly, BMR has been positively correlated with proinflammatory status in both normal and overweight individuals. This suggests that BMR may be a considerable indicator of metabolic health that is independent of obesity. In a cohort study of elderly individuals, it was found that higher BMR was associated with a greater risk of mortality, independent of BMI [25]. 

**Figure 1:**
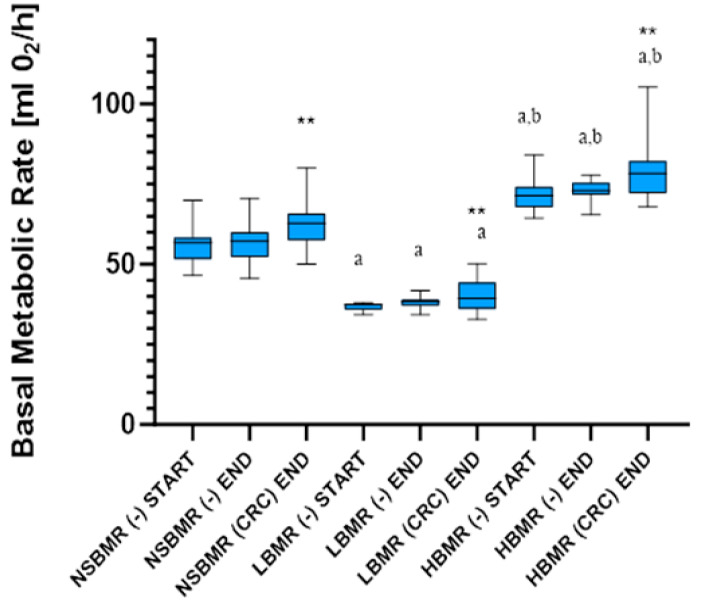
Basal metabolic rate values (start and end) in mice divergently selected for HBMR LBMR NSBMR in two experimental groups: without CRC (−) and with CRC. The results are expressed as the mean ± SEM for each group. ** *p* < 0.01 vs. animals without CRC (−) in each tested line, ^a^ *p* < 0.05 vs. NSBMR start/end (−) and (CRC) end, respectively, ^b^ *p* < 0.05 vs. LBMR start/end (−) and (CRC) end, respectively.

Our results showed that mice originating from each specific line type differed significantly with respect to the metabolic rate (ANCOVA, F_2,103_ = 717.02; *p* < 0.0001), with average BMR values of 36.35, 55.23, and 71.20 mL O_2_/h for the LBMR, NSBMR, and HBMR lines, respectively (Table 1, Figure 1). Such a marked divergence in BMR but not in body mass (F_1,103_ = 3.50, *p* = 0.83) justifies testing for associations between genetically based differentiation in energy expenditure and CRC development. Basal metabolic rate (as a mean of residual values) did not change in groups without CRC in each specific metabolic line between the beginning and the end of the experiment (*p* > 0.3 in all cases). However, the study showed that basal energy expenditure increased by about 10% during tumor development in all experimental groups (Figure 1). Thus, repeated means of body-mass-corrected BMR values were significantly affected by line type and CRC treatment (ANCOVA; F_2,100_ = 670.65; *p* < 0.0001 and F_1,104_ = 15.59; *p* = 0.0001, respectively). The BMR increase rate differs between metabolic lines, as line type x time and treatment x time interactions were significant (F_2,98_ = 4.25; *p* = 0.0169, F_1,103_ = 33.66; *p* < 0.0001, respectively). However, the insignificant interaction of line type x treatment (F_2,105_ = 1.27; *p* = 0.2854) indicates that observed variation in BMR plays a minor role in energy expenditure changes during tumor development. 

Tumor cells are characterized by an increased metabolism as a result of their proliferation and to ensure that the cells receive sufficient amounts of nutrients. They derive energy from the breakdown of high-energy adenosine triphosphate (ATP) bonds through glycolysis and the Krebs cycle, which is reflected in increased activity of the respiratory chain, leading to the formation of ROS as a result of one-electron oxygen reduction, damage to mitochondrial DNA (mtDNA), and consequently, the occurrence of, for example, increased proliferation [26]. This change is induced by an elevated absorption of nutrients, such as glucose and glutamine, resulting in a rapid rate of glycolysis, increased extracellular acidification, and the upregulation of metabolic enzymes, which can lead to changes in the body’s basal metabolic parameters [27]. Therefore, analyzing the possible mechanisms of carcinogenesis by altering the metabolic rate should be of great interest. 

### 2.2. Tumor Growth in Relation to BMR Values

Increasing evidence suggests a link between BMR and the susceptibility to developing cancer [2,24], but the exact mechanisms behind this association remain unclear. Our study, based on statistical analysis, confirmed a significant correlation between BMR values and CRC development (OR = 1.30, 95% CI 1.09–1.55, *p* = 0.0029). A higher basal metabolic rate may be associated with an increased risk of certain cancers, including colorectal, pancreatic, thyroid, postmenopausal breast, and endometrial cancers [3]. These findings suggest that BMR may serve as a useful predictor of cancer risk independent of body fatness, helping in practice to identify specific subgroups of the human population being at a higher risk of developing these types of cancers.

**Figure 2:**
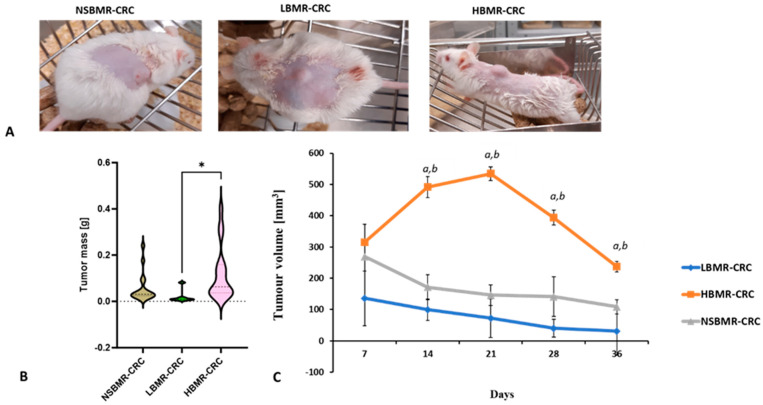
Image of tumor size (**A**), tumor mass at 36 days (**B**), and tumor growth (**C**) changes in studied groups. The results are expressed as the mean ± SEM for each group. * *p* < 0.05, ^a^ *p* < 0.01 vs. NSBMR-CRC, ^b^ *p* < 0.01 vs. LBMR-CRC.

Our study showed that the adjustment of tumors after subcutaneous application of DLD-1 cells depended on the metabolic properties of the tested mice. On the seventh day after cell implantation, we observed the development of malignant neoplasms in the form of growths in all tested mouse lines. However, the greatest tumor expansion was observed in NSBMR mice (93%) and slightly less in the HBMR group (90%), while those changes in the LBMR line were much less outlined (43.8%). The tumor growth analysis showed significantly the highest tumor volume in the HBMR line, reaching a maximum volume on the 21st day of the study (534.4 mm^3^; Figure 2A–C). It should be noted that after that time point, the size of the tumors in this group started to decrease and in the final phase of the experiment reached 237.0 mm^3^. In the NSBMR group, the initial tumor size was slightly smaller than that of the HBMR mice and gradually decreased during the study to a final size of 108.5 mm^3.^ The study also showed that tumor mass was statistically higher in the HBMR group compared to the LBMR group (*p* < 0.05), (Figure 2B). Significantly, the lowest degree of malignant tissue development during the experiment was observed in LBMR animals, and the average volume of induced tissue at the end of the study was only 31.3 mm^3^. 

**Figure 3:**
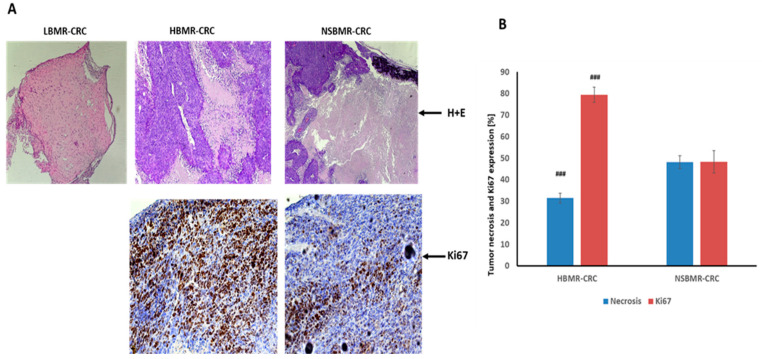
H&E staining, Ki67 expression (200× magnification) (**A**) and percentage of tumor necrosis and Ki67 expression (**B**) in mice with CRC. (No tumor cells were found by H&E staining in the LBMR-CRC group; therefore, a proliferation assay using the Ki67 antibody was not performed). ### *p* < 0.001 vs. NSBMR-CRC.

Moreover, a histopathological analysis of tissues collected from LBMR-DLD-1 mice at the end of the study did not confirm CRC induction in this group. The obtained tissues corresponded morphologically to adipose cells (Figure 3), which explains a significant reduction in tumor size in a low-BMR group. Our results clearly demonstrated, then, the contribution of metabolic rate to CRC development. Individuals characterized by high energy expenditures are more prone to promoting the induction of tumor growth compared to random-bred or low-BMR animals. 

The HBMR group showed a significant reduction in tumor volume after 21 days of tumor advancement, whereas, such growth contraction in LBMR and NSBMR lines was observed already on the 14th day of the experiment. According to the life-history theory, any significant increase in energy expenditure for processes contributing to the animal’s fitness (physiological maintenance, reproduction) can compromise immune responses [28]. It is most likely that the metabolic burden in the HBMR group has considerably reduced the intensity of immune action, resulting in the delayed inhibition of tumor growth. Studies have shown that mice with an inherited HBMR exhibit a lower immune response to antigens compared to those from the LBMR line [29]. Immunodeficient mice are known to develop more carcinogen-induced and spontaneous cancers [30]. Therefore, organisms with high metabolic rates are at a greater risk for immune deficiency and cancer development. Moreover, cancer cells are characterized by abnormal metabolism. In an aggressive tumor model, an upregulation of carbohydrate management and an increase in pyruvate and lactate production were observed in colon and breast cancer cells [31]. A high rate of glycolysis leads to the accumulation of extracellular lactate, resulting in an acidic tumor microenvironment that is conducive to metastasis, vascularization, and resistance. For example, in vitro and in vivo studies have shown that such a pH change may be beneficial to aggressive colon and breast tumors, highlighting the importance of physico-chemical conditions in cancer progression [32].

Interestingly, the metabolic characteristics of an organism also have a considerable impact on blood parameters in the tested groups of animals during CRC growth (Supplementary Table S1). The white blood cell (WBC) and platelet (PLT) counts in the HBMR-CRC group were significantly higher than those in the NSBMR and LBMR groups (CRC and without CRC groups) (*p* < 0.05). This demonstrates that these animals experienced the greatest development of an inflammatory condition, which resulted in the strongest tumor growth. Clinical studies have shown that an increase in platelet count is associated with an advanced stage of cancer and is an unfavorable prognostic marker [33]. Changes in the number of cancer-related blood cells, followed by metabolic rate adjustment, indicate stronger inflammatory status and more aggressive tumor development in individuals with higher BMR. 

### 2.3. Effect of BMR on CRC Development and Ki67 Expression

The histopathological analysis revealed significant differences in the characteristics of CRC development and growth in the tested mouse lines (Figure 3A). The H&E test clearly indicated the development of CRC in the harvested tumors in the HBMR-CRC and NSBMR-CRC groups. G3-type poorly differentiated colorectal neoplasia masses were found in both metabolic lines. However, the analysis of tissues from LBMR-CRC mice did not confirm CRC structures and morphologically pointed rather to something resembling fibrous connective tissue that underwent a significant hyalinization process. Due to the volume reduction of the CRC tumors during the experiment, the degree of necrosis in the collected tissues was also estimated. Neoplasm necrosis is one of the prognostic indicators, particularly in aggressive tumors [34]. Accordingly, it has been suggested that a higher degree of necrosis may indicate more aggressive tumor growth and, thus, a worse prognosis for the patient [35]. In addition, necrosis is often associated with an inflammatory response aimed at removing dead cells. The analysis of identified tumors showed that in the HMBR-CRC group, the degree of tumor necrosis was estimated at 31.4%, whereas in the NSBMR-CRC group, it was significantly higher (48.1%) (Figure 3B). As was discussed above, the significant decrease in tumor volume observed in high-BMR mice after the 21st day of the experiment may be attributed to a necrotic process.

The expression of Ki67 is strictly associated with cell proliferation. Ki67 protein is present in all active phases of the cell cycle (G1, S, G2, and mitosis); therefore, it is the best marker to determine the growth of cell population, and likewise, it can indicate growing tumor cells [36]. Our results showed a positive reaction of Ki67 in all identified CRC tumors. Nonetheless, significantly the highest expression of Ki67 was observed in the HBMR-CRC group (79.4%), whereas the NSBMR-CRC group of mice had an estimated expression of about 48.3%. DLD-1 is a colorectal adenocarcinoma cell line, Dukes’ type C, characterized by a high degree of proliferation, thus indicating a high degree of malignancy of this tumor. Previous studies with an analysis of Ki67 expression on 1800 CRC samples showed that a low Ki67 index is associated with a low tumor stage and is an independent prognostic factor for favorable survival [37]. The lack of CRC indicators in mice with a low-BMR status at the end of the experiment and significantly marked Ki67 expression in high-BMR individuals suggest an increased susceptibility to colon cancer development.

### 2.4. Oxidative and Antioxidant Status in Experimental Animals

**Figure 4:**
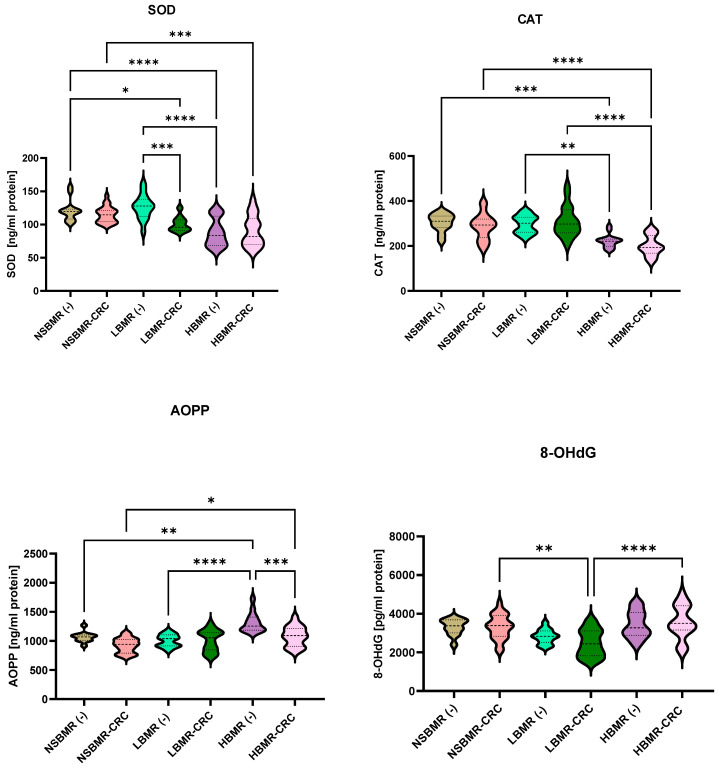
SOD, CAT, AOP, and 8-OHdG concentrations in livers of the studied animal groups. The results are presented as violin plots for each group. Differences statistically important: * *p* < 0.05, ** *p* < 0.01, *** *p* < 0.001, **** *p* < 0.0001.

It is widely recognized that a high basal metabolic rate requires more energy to maintain organismal homeostasis. ROS are toxic by-products of cellular energy metabolism that can cause the oxidation of proteins and nucleic acids and contribute to cellular aging and carcinogenesis [2,9]. BMR is positively correlated with intracellular ROS production in organisms. Therefore, for further investigation, we analyzed the concentrations of superoxide dismutase (SOD) and catalase (CAT), being important antioxidant enzymes that act as a first-line defense [38], and the oxidative proteins advanced oxidation protein products (AOPP) and 8-hydroxy-2′-deoxyguanosine (8-OHdG) in the livers of the studied animals. AOPPs are considered the main, ROS-related biomarkers of oxidative stress in various inflammatory diseases [21]. Moreover, 8-OHdG in nuclear and mitochondrial DNA is a common form of free-radical-induced oxidative lesions and is widely used as a biomarker for oxidative stress and carcinogenesis [39]. Our results showed the differences in SOD and CAT concentrations in liver homogenates among the tested groups (Figure 4). The LBMR(−) animals had the highest SOD concentration (126.3 ng/mL protein), while the lowest concentration was observed in the group with HBMR(−) animals (87.9 ng/mL protein) (*p* < 0.0001). In the case of CAT analysis, the control groups with the highest concentration were the NSBMR(−) and LBMR(−) animals (304.0 and 297.8 ng/mL protein, respectively), while the HBMR(−) group had the significantly lowest concentration (mean of 222.3 ng/mL protein). 

The analysis of oxidative status in the livers of animals without CRC clearly showed that the highest AOPP concentration was exhibited by mice with high BMR (1311.0 ng/mL protein) compared to LBMR (1006.0 ng/mL protein). Our study demonstrated that the concentration of 8-OhdG was significantly higher in the HBMR(−) and NSBMR(−) groups (3437.0 and 3314.0 ng/mL protein, respectively) compared to LBMR(−) animals (2818.0 ng/mL protein). The development of CRC resulted in a significant decrease in CAT concentration in HBMR-CRC mice (201.5 ng/mL protein) compared to NSBMR-CRC (286.9 ng/mL protein, *p* < 0.0001) and LBMR-CRC (309.0 ng/mL protein, *p* < 0.0001). A significant (*p* < 0.001) decrease in SOD concentration was noticed in the LBMR-CRC group compared to the LBMR(−) mice. It is important to note that the development of CRC resulted in a slight increase in 8-OhdG in HBMR-CRC and NSBMR-CRC mice when compared to their respective groups without CRC. Furthermore, AOPP levels were found to be significantly reduced in the HBMR-CRC group compared with those of the HBMR(−) animals and were found to be significantly higher compared with those of the NSBMR-CRC group. 

**Figure 5:**
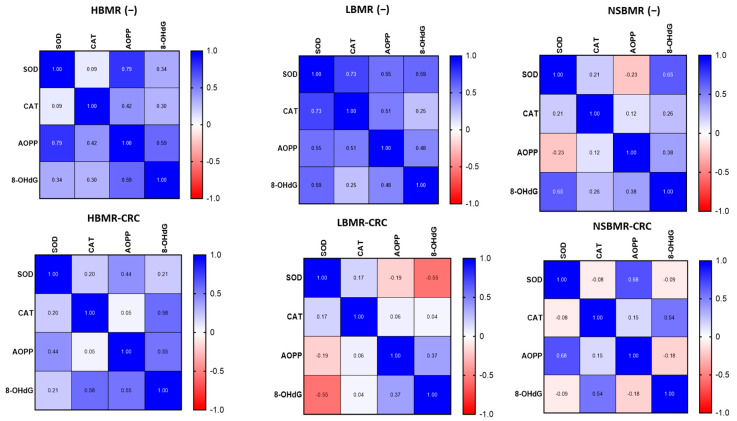
The Pearson correlations of antioxidant/oxidative enzyme levels in livers of the studied animal groups. The results are presented as heat maps with r values for each group.

The analysis of antioxidant and oxidant status in the liver of the experimental groups showed a high correlation between 8-OhdG and AOPP (r = 0.59) but a weak relation between SOD and CAT (r = 0.09) in HBMR(−) animals (Figure 5). On the other hand, the LBMR(−) group exhibited a positive correlation between SOD and CAT concentrations, indicating a high antioxidant status in these animals (r = 0.73). In the NSBMR(−) group, we noted a correlation between 8-OhdG and AOPP (r = 0.38) and between SOD and CAT (r = 0.2). The presented results suggest a high oxidative status in the livers of HBMR(−) mice, while a high antioxidant status was attributed to the LBMR(−) group. 

**Figure 6:**
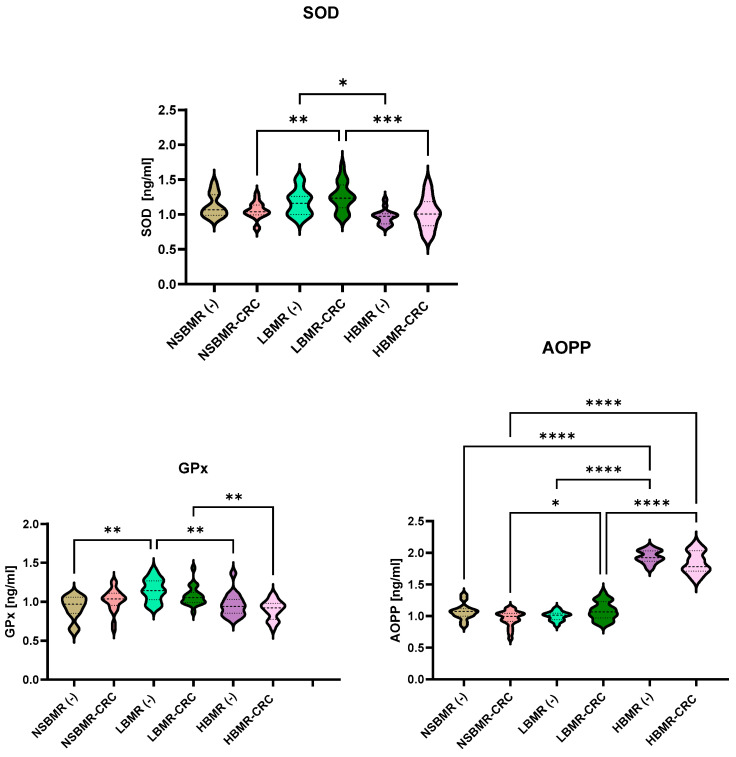
SOD, GPx and AOPP concentrations in serum of the studied animal groups. The results are presented as violin plots for each group. Differences statistically significant: * *p* < 0.05, ** *p* < 0.01, *** *p* < 0.001, **** *p* < 0.0001.

**Figure 7:**
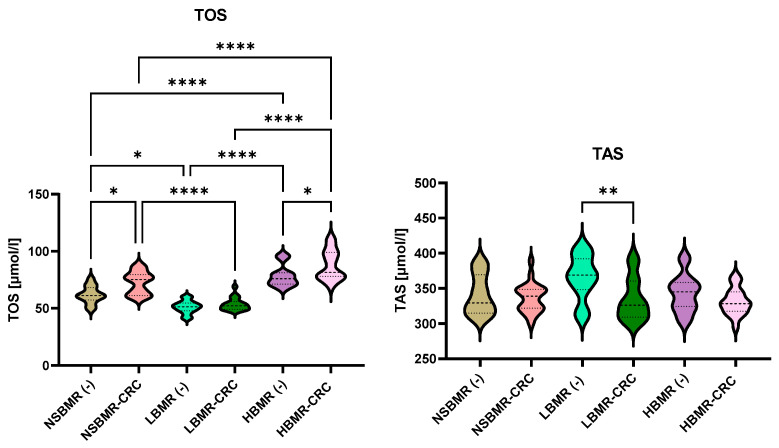
TAS and TOS concentrations in serum of the studied animal groups. The results are presented as violin plots for each group. Differences statistically significant: * *p* < 0.05, ** *p* < 0.01, **** *p* < 0.0001.

**Figure 8:**
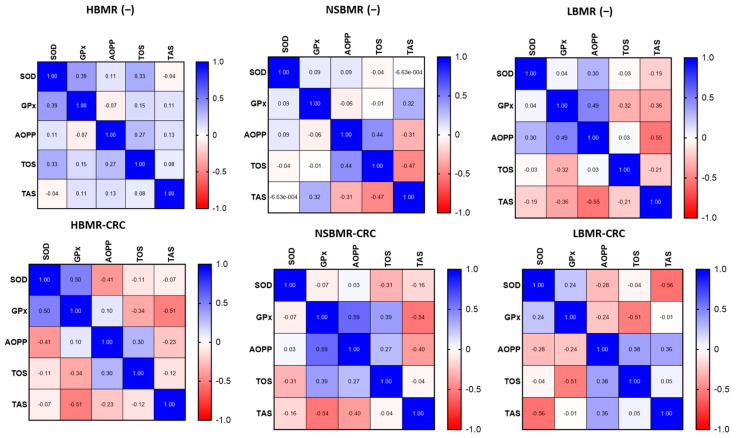
The Pearson correlations of antioxidant/oxidative enzyme levels in serum of the studied animal groups. The results are presented as heat maps with r values for each group.

Furthermore, an analysis of the total oxidative (TOS) and antioxidant (TAS) statuses of the serum of the tested animal groups revealed significant differences in values between the tested animal lines (Figure 6, Figure 7 and Figure 8). The results showed that HBMR mice both without and with CRC had the highest levels of TOS (respectively: 78.4 µmol/mL and 87.9 µmol/mL), AOPP (respectively: 1.9 ng/mL and 1.8 ng/mL) in their serum, compared to NSBMRand LBMR animals. Furthermore, the concentration of TOS in the experimental groups with CRC cells in all tested animal lines was significantly higher than in their respective groups without CRC(−). Regarding antioxidant status, the highest level of TAS was detected in the LBMR group without CRC (365.6 µmol/mL), which is also related to the high concentration of antioxidant enzymes such as SOD (1.2 ng/mL) and glutathione peroxidase (GPx) (1.1 ng/mL) in the tested animal line. 

The present results clearly showed that HBMR mice are characterized by higher oxidative status compared to animals with low or intermediate basal metabolic rates. It has been shown that the body-mass-independent variation in MR of organisms can be mainly attributed to cell size differentiation [40]. Genetically determined differences in basal metabolism in the used model (Table 1) are a result of the heterogeneity of their internal architecture. Individuals with high BMR are characterized by substantially larger metabolically active organs followed by an increase in the size of cells specific for those tissues [13,19] compared to animals with low MR. This suggests that the substantial contribution of metabolic components operating at a higher rate of cellular metabolism in high-BMR individuals generates higher concentrations of ROS, resulting in an increased risk of cancer development in these animals. So far, studies have suggested that oxidative stress plays a significant role in the development of cancerogenic changes, including CRC [11,12]. Several studies have reported increased ROS concentrations and decreased antioxidant mechanisms in CRC, although the data are not conclusive [41]. Our studies showed that CRC line DLD-1 development led to a significant increase in oxidation status, notably in the HBMR and NSBMR groups. Firstly, an altered ROS concentration in individuals with high energy turnover may simply be a consequence of the overintensity of oxygen-related processes in metabolic active organs like the liver. Secondly, cancer cells themselves can increase the ROS concentration in the body, which is associated with neoplasm growth and metastasis. Tong et al. [11] demonstrated that the level of ROS was higher in sporadic-type CRC in comparison to normal cells, which may be connected with the increased metabolic activity of colorectal cancer. Furthermore, cancer cells have strong antioxidant defenses to adapt to elevated ROS and avoid apoptosis [42]. The increase in CAT concentration in the livers of NSBMR-CRC and HBMR-CRC mice in comparison to their groups without CRC can explain the increase in antioxidant activity that occurs in the initial stages of colon cancer development. Moreover, it was shown that the expression of antioxidant-related enzymes, specifically SOD, increased in stages III and IV of CRC tumor samples, while the expression of other antioxidant enzymes, such as catalase and GPx, increased in adjacent non-tumor tissues [43]. Similar changes in SOD activity levels were observed in advanced stages of CRC, lung cancer, and gastric carcinoma [44,45]. A consequence of the observed rise in the activity of antioxidant enzymes is a decline in the concentration of oxidative enzymes. A significant decrease in AOPP concentration in HBMR-CRC animals was observed. AOPP is defined as dithyrosine-containing cross-linked protein products and is regarded as a reliable marker for quantifying the extent of oxidant–protein damage [46]. These products are the result of the action of free radicals on proteins and may act as inflammatory mediators, triggering the oxidative ignition of neutrophils, monocytes, and T-lymphocytes. A reduction in the levels of AOPP may therefore indicate a stimulation of the immune and antioxidant response, which in turn leads to a stimulation of the mechanisms that prevent DNA damage and the development of pathological processes, including cancer [47]. Thus, undoubtedly, high BMR may increase the risk of tumor development. 

### 2.5. Keap1 Expression in the Liver of Experimental Animals

**Figure 9:**
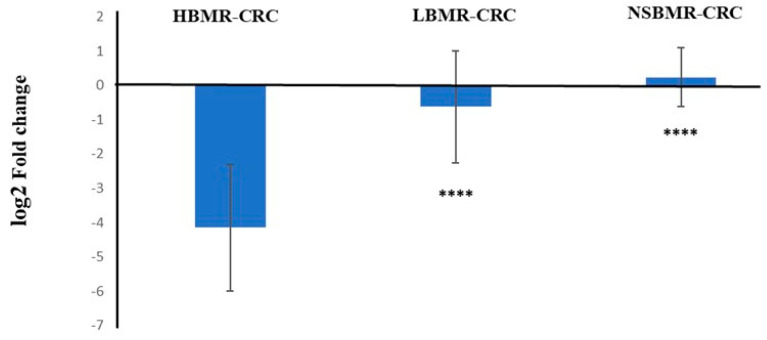
*Keap1* expression in livers of studied animal groups with CRC. The results are presented as log2 delta-corrected Ct values. Differences statistically significant: **** *p* < 0.001 vs. HBMR-CRC group.

To counteract oxidative damage, the body has an efficient defense system that detoxifies and eliminates harmful chemicals and includes the inactivation of ROS. *Nrf2* serves as a master regulator of multiple antioxidant enzymes, modulating cell redox balance and sensing the status of cellular oxidative stress. This can be achieved by stimulating the activity of antioxidant defense components, such as SOD, GPx, and CAT [23]. Under oxidative stress, *Keap1* acts as a master negative regulator of *Nrf2*. It undergoes a conformational change that causes *Nrf2* to dissociate and aggregate. *Nrf2* then combines with antioxidant response elements (AREs) to initiate the transcription of multiple antioxidants [48]. In our research, we indicated that expression of the *Keap1* gene in the liver tissue was considerably downregulated in HBMR-CRC animals (log2 fold change: −4.15), but remains unchanged in LBMR-CRC and NSBMR-CRC groups (log2 fold changes: −0.63 and 0.24, respectively) (Figure 9). 

The reduction in AOPP levels and increase in CAT in the livers of HBMR-CRC animals is largely explained by the low expression of *Keap1*. On the other hand, the unchanged expression of *Keap1* in the low-BMR animals, along with high concentrations of antioxidant enzymes SOD, CAT, and TAS, may explain the reduced intensity of oxidative stress and the lack of CRC tumor induction in mice treated with DLD-1 cells. The exact mechanism through which *Keap1* influences tumorigenesis and development is still unclear. The protein Keap1 forms a complex with the ubiquitin ligase E3, cullin-3, which subsequently binds to the transcription factor *Nrf2*, a key regulator of cellular oxidative stress. Theantioxidant responsive element (ARE) signaling pathway is regulated by the ubiquitination and degradation of Nrf2, affecting anti-oxidation proteins that subsequently influence tumor cell proliferation, apoptosis, and sensitivity [49]. A low expression of *Keap1* is associated with various malignancies, including non-small cell lung cancer, colorectal cancer, liver cancer, gallbladder cancer, breast cancer, and head and neck squamous cell carcinoma [45,50]. Our results suggest that DLD-1 tumor growth causes a decrease in the *Keap1* gene expression in HBMR-CRC animals compared to the LBMR and NSBMR lines. This indicates that *Keap1* functions as a tumor suppressor, and its decreased expression in HBMR animals is associated with CRC development. Moreover, it has been demonstrated that some cancers exhibit constitutively high levels of *Myc* Proto-Oncogene (*c-Myc*) expression, including cases of colorectal cancer or non-small cell lung cancers, and it is possible that the reduction in Nrf2 occurs as a result of increased *c-Myc* [51,52]. 

Mammalian targets of rapamycin (mTOR)-related cellular activity constitute an important pathway that may directly interact with *Keap1* and NRF2. mTOR is a key factor involved in many cellular processes that regulate intracellular signaling molecules providing energy supply to the cell [53]. In particular, mTOR facilitates the nuclear translocation of *Nrf2* by inhibiting the expression of beta-transducin repeats-containing proteins *(β-TrCP)* and participates in the signaling of *Keap1*. Nonetheless, the reduced expression of *Keap1* correlates with the increased activity of *Nrf-2,* which is also associated with the increased expression of mTOR [54]. Our previous study revealed that HBMR mice have a significantly higher expression of the mTOR gene in hepatocytes compared to LBMR mice [5]. High levels of mTOR are also observed in many tumor types, suggesting that the over-expression of this protein may be one of the key mechanisms triggering cancer [53]. The involvement of the mTOR signaling pathway in the development of CRC is well documented [55]. Simultaneously, the inhibition of mTOR signaling has been shown to suppress CRC cell growth in vitro and adenoma formation in vivo [56]. As the mTOR protein contributes to both metabolic and cancer-related processes, it provides further evidence that organisms with inheritably high metabolic rates are characterized by a higher risk of cancer development.

Our results showed that oxidative/antioxidative conditions are significant for the prevention/initiation of DLD-1 colorectal cancer development. Moreover, the activation of *Keap1-Nrf2* also contributes to the stimulation of mechanisms associated with tumor progression. Therefore, preventive treatment that attains *Nrf2* activation seems to reduce cancer initiation and would be of particular value for those who are at high risk of CRC cancers. Overall, this study revealed that oxidative stress and the deactivation of *Keap1-Nrf2* are important in CRC development. 

## 3. Materials and Methods

### 3.1. Animals and Experiment Design

In this study, we used 22-week-old males of laboratory mice (*Mus musculus*) originating from three genetic lines. Two of those lines were subjected to divergent nonreplicated artificial selection either towards high or low body-mass-corrected BMR (for details, see Książek et al. [57]). The third line represented randomly bred animals as part of a concurrent artificial selection experiment (NSBMR). Following BMR measurements, animals from each line type were randomly assigned to one of the experimental groups (day 0): standard control (without CRC (−)) and group with implemented DLD-1 cells (named with CRC) (N = 12 for each group and line type). BMR measurements were repeated in all animals three days before the end of the study to assess changes in metabolic rate from the beginning to the end of the experiment. On the 35th day of the experiment, the animals were anesthetized and then sacrificed by intraperitoneal administration of a mixture of ketamine and xylazine at doses of 50 mg/kg and 5 mg/kg, respectively. Blood and organs were taken for further biochemical and genetic analysis. The morphological examination of blood collected from the animals was performed using the ABCvet system. Animals were housed at 23 ± 0.1 °C and 12d:12n photoperiod with unlimited access to water and food.

All procedures were carried out by the European Directive (2010/63/EU). Handling of animals was performed with the consent of protocols approved by the Local Ethical Committee in Olsztyn no 68/2020.

### 3.2. Basal Metabolic Rate Measurements

Briefly, mice were individually placed in 350 cm^3^ chambers that were immersed in a water bath set to a temperature of 32 °C (thermoneutral zone). The air streams, powered by separate mass flow controllers at a rate of 400 mL min^−1^, were pushed through the chambers, cleared of moisture and CO_2_, and directed to the Sable Systems TR-1 oxygen analyzer (Henderson, NV, USA). The air was sampled at a rate of 75 mL min^−1^. Measurement of metabolism lasted approximately 3 h, with the first 60 min set for acclimatization. Oxygen concentration in each chamber was assessed every second during the 2 h trial. BMR was defined as the lowest level of oxygen consumption that did not change by more than 0.01% for at least 4 min. During the measurement, the performance analysis was based on Sable System DATACAN V *Statistica* 12.0 software (StatSoft, Tulsa, OK, USA).

### 3.3. Cell Culture and Induction of Colon Cancer

Studies were conducted with DLD-1 colon cancer cell line purchased from American Type Culture Collection, (Manassas, VA, USA). The cells were maintained in Dulbecco’s Modified Eagle Medium (Gibco) supplemented with 10% fetal bovine serum (Gibco) and antibiotics (Gibco) in a humidified incubator at 37 °C and 5% CO_2_ atmosphere. Sub-confluent cells were detached with Trypsin solution (Gibco) in calcium-free phosphate-buffered saline (PBS) (Corning) and counted in hemocytometer. Animals assigned to experimental groups were implanted subcutaneously with DLD-1 cells (2 × 10^7^) in 0.1 mL volume of Matrigel/PBS (Alchem, Poland). To keep the test pattern, the remaining 12 mice of each group (HBMR, LBMR, and NSBMR) were administered subcutaneously with PBS. The degree of proliferation of CRC was defined as the volume of induced tissue and was measured twice a week (every 3–4 days) via a digital caliper and calculated using the following formula: (0.52 × width^2^ × length).

### 3.4. Histopathological and Immunohistochemical Analysis 

Tissue sections were taken during the autopsy, fixed in buffered formalin solution, and embedded in paraffin. The paraffin blocks were then sliced with a microtome into 4 µm thick sections, placed on salinized slides, and stained with hematoxylin and eosin (H&E). The histopathological examination evaluated the percentage of necrotic area in relation to tumor area with the use of Olympus, Tokyo, Japan, EP50 camera. For immunohistochemical analysis, the slides were incubated overnight at 60 °C and then deparaffinized in xylene solutions and rehydrated in a series of alcohols in concentrations of 99.9%, 96%, and 70%. Next, the sections were placed in citrate buffer (pH = 6.0) and incubated in a water bath for 20 min at 98.5 °C to reveal the antigen and then incubated for 20 min at room temperature. Endogenous peroxidase was blocked by using 3% hydrogen peroxide for 10 min, followed by 2.5% goat serum, to block nonspecific antibody binding (Vector Laboratories, Eching, Germany) for 10 min. Then, the slides were incubated with specific antibodies Ki67 (Biorbyt, dilution 1:100) for 30 min at room temperature. Antibody-binding sites were visualized using the ImmPress Goat Anti-Rabbit IgG Polymer Reagent kit (Vector Laboratories, Eching, Germany) for 30 min and ImmPACT DAB chromogen (Vector Laboratories, Eching, Germany) for 5 min. Cell nuclei were stained with hematoxylin, and slides were dehydrated in a series of alcohols of increasing concentrations of 70%, 96%, and 99.9% and washed in xylene solutions. The evaluation of immunohistochemical staining was performed with a light microscope using 200× magnification. For H&E and immunohistochemical experiments, we used 6–10 mice per group. Digital images were acquired from 10 different fields in each field of view. The 100 cancer cells were assessed. The percentage of necrosis and Ki67 proliferation was estimated in the field of view of each section and averaged for each group.

### 3.5. Preparation of Homogenates

Liver homogenates were prepared from harvested animal tissues previously frozen in liquid nitrogen and stored at −80 °C. First, the tissues were rinsed in ice-cold PBS to thoroughly remove excess blood and weighed before homogenization. Next, the liver tissues were homogenized on ice in fresh lysis buffer (Cell Biologics, Chicago, IL, USA) and proteolysis inhibitor (1 tablet/10 mL buffer, Complete Mini Roche, Basel, Switzerland) at a ratio of 1:20. The resulting suspension was sonicated with an ultrasonic cell disrupter and then centrifuged (10 min, 10,000× *g*, 4 °C). A supernatant was retained for further studies and used for biochemical assays. The total protein concentration in liver homogenates was determined using the commercial BCA^TM^ Protein Assay Kit (Pierce, Thermo Fisher Scientific, Rockford, IL, USA).

### 3.6. Biochemical Assays

The biochemical assays of the SOD, CAT, AOPP, and 8-OHdG concentrations in livers were determined using the commercial Enzyme-linked Immunosorbent Assay Kit for mice (Cloud-Clone Corp. Unit, Katy, TX, USA). Additionally, TAS and TOS were determined in serum using the ImAnOx (TAS/TAC) Kit and PerOx (TOS/TOC) Kit (Immundiagnostik, Bensheim, Germany). SOD, GPx, and AOPP concentrations in serum were analyzed using the commercial Enzyme-linked Immunosorbent Assay Kit for mice (Cloud-Clone Corp. Unit, Katy, TX, USA). The absorbance was measured using a microtiter plate reader (BioTek Epoch, Hopkinton, MA, USA). All results were standardized to 1 mg of total protein in the livers and serum.

### 3.7. Keap1 Expression

The total tissue RNA was isolated from the liver samples with a standard commercial kit (Total RNA mini plus, A&A Biotechnology, Poland). DNAse treatment with a Clean-up RNA concentrator (A&A Biotechnology, Gdansk, Poland) was applied to remove traces of DNA from all samples. RNA purity was checked using the NanoDrop2000 (ThermoFisher). Primer-specific, high-capacity cDNA reverse transcription kit was used to convert RNA to cDNA (TranScriba kit, A&A Biotechnology, Poland). All RNA samples were adjusted to a starting concentration of 50 ng/μL. The expression levels of *Keap1(For 5′-AGCCAGCAACTCTGTGACG and Rev 5′-GAACCACGCTGTCAATCTGG)* and *β-actin* genes (as a reference) were estimated with SYBR Green (RT PCR MixSYBR, A&A Biotechnology, Poland) on real-time PCR (Applied Biosystems). ΔCt values for mKeap1 in experimental groups were corrected for untreated groups (without CRC) with average ΔCt as the calibrator, according to the 2^−ΔCt^ method [58]. 

### 3.8. Statistical Analysis

Statistical analysis was performed using Statistica 12.0 (StatSoft, Tulsa, USA) and GraphPad Prism 9.0 (GraphPad Software, Boston, MA, USA). BMR was analyzed by analysis of covariance, with line type and experimental group as fixed factors and body mass as a covariate. The differences in body mass and blood parameters were analyzed with a two-way analysis of variance, with the line type and experimental group as fixed factors. Intergroup statistical comparisons were analyzed using standard statistical analyses, including one-way analysis of variance (ANOVA), followed by post hoc Duncan’s or Tukey’s comparison test. Differences were considered significant when *p* < 0.05. The relationship between the quantitative data was assessed according to Pearson rank correlation and presented as heat maps. 

## 4. Conclusions

In conclusion, our study suggests that the genetically determined metabolic properties of organisms may stand as an important risk factor in the development and progression of colorectal cancer. Differences in BMR directly translate into the characteristics of cancerogenesis processes, including cell proliferation and growth. We provide evidence that DLD-1 colorectal cancer grows more rapidly in mice characterized by higher energy expenditures, which is associated with differences in ROS levels, the antioxidant barrier, and Keap1 expression. Here, our study offers novel insights into the correlation between metabolic rate and DLD-1 colorectal cancer. The obtained results may guide medicine towards the combination of energy metabolism inhibitors with other anti-cancer drugs as a new and effective approach to the treatment of colorectal cancer.

## Figures and Tables

**Table 1:** The comparison of phenotypic traits in mice divergently selected for high and low BMR and NSBMR mice in two experimental groups: treated with CRC and without CRC (−).

Parameters	Line Type						GLM Analysis	
	NSBMR		LBMR		HBMR		Line Type	
	(−)	CRC	(−)	CRC	(−)	CRC	F_2,98_	*p*
Body Mass [g]	39.35 ± 4.41	38.30 ± 3.26	34.36 ± 2.04	34.09 ± 3.18	34.30 ± 2.55	35.26 ± 2.32	23.50	<0.001
Body Mass END [g]	40.42 ± 4.93	38.84 ± 2.74	37.13 ± 2.59	33.63 ± 3.25	35.93 ± 1.98	36.94 ± 3.02	16.63	<0.001
BMR [mL O_2_/h]	52.88 ± 5.99	-	38.21 ± 2.36 ^a^	-	71.01 ± 4.71 ^a,b^	-	717.02	<0.001 ^(+)^
BMR END [mL O_2_/h]	53.15 ± 5.02	62.10 ± 6.28	37.64 ± 1.85 ^a^	42.26 ± 4.61 ^a^	72.15 ± 3.92 ^a,b^	76.85 ± 6.17 ^a,b^	670.65	<0.001 ^(+)^

Effects of line type (HBMR, LBMR, NSBMR) and CRC treatment tested with general linear models. Directional relationship with a body mass (covariate) is indicated by a + sign (if significant) together with *p*-values. Significant differences in mean values between line types (Tukey test) are indicated by different letters (^ab^). ^a^ *p* < 0.05 vs. NSBMR start/end (−) and (CRC) end, respectively, ^b^ *p* < 0.05 vs. LBMR start/end (−) and (CRC) end, respectively.

## Data Availability

The data supporting the findings of this study may be obtained upon reasonable request from the corresponding author.

## Supplementary Materials

The following supporting information can be downloaded at: https://www.mdpi.com/article/10.3390/ijms251910713/s1.
