# Supplementary material for: Metabolic Rate and Oxidative Stress as a Risk Factors in the Development of Colorectal Cancer
Source: Int J Mol Sci. 2024 Oct 5;25(19):10713. doi: 10.3390/ijms251910713 (PMC11476475; doi:10.3390/ijms251910713)
Supplement: Supplementary file 1 [file ijms-25-10713-s001.zip › ijms-3237081-supplementary.pdf]

Supplementary Materials

Table S1. Morphological blood parameters of control and experimental mice.

Parameters	NSBMR		LBMR		HBMR	
	(-)	CRC	(-)	CRC	(-)	CRC
**WBC [10^3/mm^3]**	1,76 ± 0,30	2,00 ± 0,63	2,34 ±0,69	1,73 ± 0,59	2,79 ± 0,95 [d]	4,38 ±1,59 [a,b,c,d]
**RBC [10^6/mm^3]**	9,36 ± 2,09	8,28 ± 2,14	8,39 ± 1,20	7,99 ± 1,99	8,10 ± 2,11	7,79 ±1,48
**HGB [g/dl]**	14,88 ± 2,75	12,91 ± 3,36	12,81 ±1,60	12,87 ± 2,69	12,59 ± 2,70	11,84 ± 2,11[a]
**HCT [%]**	52,48 ± 9,46	44,75 ± 12,0	42,89 ± 5,83	41,82 ± 9,73	43,2 ± 10,55	40,53 ±8,65
**MCV [μm^3]**	54,09 ± 2,21	54,45 ± 1,74	51,56 ± 1,90	51,28 ± 1,56	52,62 ± 1,78	52,84 ±1,80
**MCH [pg]**	15,57 ± 0,64	15,80 ± 1,73	15,02 ± 0,56	15,24 ± 0,77	15,47 ± 1,43	15,45 ±0,61
**MCHC [g/dL]**	28,74 ± 0,48	29,07 ±2,87	25,59 ± 0,39	29,88 ± 1,59 [c]	28,73 ± 0,87 [c]	29,29 ±1,25 [c]
**PLT [10^3 /mm^3]**	611,73 ± 141,80	572,23 ±183,25	457,37 ± 120,42	265,00 ± 133,63 [a,b]	642,25 ±231,86 [d]	966,64 ±317,05 [a,b,c,d,e]

The results are presented as the mean values + SD. [a] $p<0.05$ vs. NSBMR(-), [b] $p<0.05$ vs. NSBMR-CRC, [c] $p<0.05$ vs. LBMR(-) , [d] $p<0.05$ vs. LBMR-CRC, [e] $p<0.05$ vs. HBMR(-) **Abbreviations:** WBC white blood cells, RBC red blood cells, HGB hemoglobin concentration, HCT hematocrit, MCV mean corpuscular volume, MCH mean corpuscular hemoglobin, MCHC corpuscular/cellular hemoglobin concentration, PLT platelet
